# Adapt-A-Maze: An Open-Source Adaptable and Automated Rodent Behavior Maze System

**DOI:** 10.1523/ENEURO.0138-25.2025

**Published:** 2025-06-17

**Authors:** Blake S. Porter, Jacob M. Olson, Christopher A. Leppla, Éléonore Duvelle, John H. Bladon, Matthijs A. A. van der Meer, Shantanu P. Jadhav

**Affiliations:** ^1^Neuroscience Program, Department of Psychology, and Volen National Center for Complex Systems, Brandeis University, Waltham, Massachusetts 02453; ^2^Department of Psychological and Brain Sciences, Dartmouth College, Hanover, New Hampshire 03755

**Keywords:** behavior, cognition, maze, open-source, rodent navigation

## Abstract

Mazes are a fundamental and widespread tool in behavior and systems neuroscience research in rodents, especially in spatial navigation and spatial memory investigations in *ad libitum* behaving animals. However, their form and inflexibility often restrict potential experimental paradigms that involve multiple or adaptive maze designs. Unique layouts often lead to elevated costs, whether financially or in terms of time investment from scientists. To alleviate these issues, we have developed an automated, modular maze system that is flexible and scalable. This open-source Adapt-A-Maze (AAM) system will allow for experiments with multiple track configurations in rapid succession. Additionally, the flexibility can expedite prototyping of behavioral paradigms. Automation ensures less variability in experimental parameters and higher throughput. Finally, the standardized componentry enhances experimental repeatability within labs and replicability across labs. Our maze was successfully used across labs, in multiple experimental designs, with and without extracellular or optical recordings, in rats. The AAM system presents multiple advantages over current maze options and can facilitate novel behavior and systems neuroscience research.

## Significance Statement

We have developed an open-source, modular maze system (the Adapt-A-Maze, AAM) that enables any lab interested in rodent behavior and cognition to create standard and unique mazes for their research. The AAM uses modular track pieces that can be combined to create a wide array of maze types. The AAM system also included reward wells with lick detection and pneumatic barriers. All aspects of the maze can be controlled via TTL signals to automate tasks and improve repeatability of experiments. The AAM system is expected to help labs quickly and inexpensively set up recordable experiments to advance our understanding of the neurophysiology underlying behavior and cognition.

## Introduction

Rodents navigating mazes is a long-standing and widespread method for investigating behavior, cognitive processes, and neurophysiology (for review, see Wijnen et al., 2024). Mazes leverage the fact that navigating to rewards or away from danger is a natural task for both rats and mice. Beginning over a century ago at the advent of the twentieth century (Small, 1901), maze experiments have resulted in landmark discoveries regarding cognitive processes of learning and memory underlying navigation (Tolman et al., 1946) and its neural substrates (O’Keefe and Dostrovsky, 1971; Olton and Samuelson, 1976). Currently, standard maze designs are pervasive and underlie foundational tasks in learning and memory, decision-making, and even anxiety (O’Keefe and Dostrovsky, 1971; Olton and Samuelson, 1976; Barnes, 1979; Morris et al., 1982; Handley and Mithani, 1984; Frank et al., 2000). In addition, unique mazes are constantly being designed to test specific questions in these fields (Knierim et al., 2000; Nitz, 2006; Ainge et al., 2007; Steiner and Redish, 2014; Wilson et al., 2015; Olson et al., 2017; Porter et al., 2018; Tanila et al., 2018; Böhm and Lee, 2020; Wijnen et al., 2024). Looking forward, the ability to rapidly switch environments while monitoring neural activity continuously is a growing desire in systems neuroscience to investigate contextual memory and representations, spatial remapping, and flexible decision-making (Karlsson and Frank, 2009; Huelin Gorriz et al., 2023; Awh et al., 2024). The explosion of neural data recording capabilities is supported by the advances in machine learning techniques for analysis, giving increasing value to complex behavioral datasets.

Current maze designs limit the ability for multiple maze environment recordings as part of one experiment. Simple mazes often consist of one piece, with limited or no flexibility for other maze configurations. If multiple maze configurations are desired, they must all be made, stored, and then moved into and out of place in the room. This limits potential experiments and slows research progress. Alternatively, scientists will spend months engineering complex, automated mazes to address a specific question. Despite the time invested, these mazes are not often amenable to flexibly changing into other maze configurations, and so the same cost in design and manufacturing must be incurred again for the next experiment.

To address these issues, we have developed the Adapt-A-Maze (AAM), an open-source automated maze system that uses standardized track pieces to create flexible and scalable maze environments for rodents. The maze can be rapidly adapted to facilitate experiments that use multiple configurations, switching between shapes on the scale of minutes. Standardized reward wells are integrated into the track pieces and support automated lick detection and reward delivery along with quick, effective cleanup. Automated movable barriers can be placed between any track components, allowing additional environmental manipulations even during active behavior. Careful consideration of component selection enables artifact-free electrophysiological recordings. Being open source, labs across the world could more easily replicate the experiments of other labs using the AAM. The AAM will allow researchers to complete better experiments faster, with greater repeatability, and at lower cost, supporting the advance of our understanding of rodent behavior and neurophysiology.

## Materials and Methods

### Materials availability

All resources are made available on our Gitlab repository: https://gitlab.com/jadhavlab/AdaptAMaze/-/tree/main?ref_type=heads. This includes schematics, 3D printer design files and print files, track piece sheet metal SolidWorks files, printed circuit board (PCB) files, SpikeGadgets ECU scripts for automating simple tasks, and a detailed parts list. We have endeavored to use, or at least publish, file types that can be opened and modified in a free software. Our goal is to make it so anyone can clone our repository and get their own AAM running in a few weeks. We look forward to users making modifications, creating new track pieces, and developing additional components to add to the repository.

### Code accessibility

We have provided some example Spikegadgets Statescript programs can be found in the repository (https://gitlab.com/jadhavlab/AdaptAMaze/-/tree/main/Example%20Statescript%20programs?ref_type=heads).

### AAM methods

Our parts list has detailed ordering and fabrication notes on applicable parts. Figure 2 and Extended Data Figure 2-1 show the schematics of a full system. Extended Data Figures 3-1, 3-2, and 4-1 show detailed pictures of all components and their part IDs. Part IDs and Excel sheets are color coded by the system they belong to.

#### Track pieces

**Figure 1.**
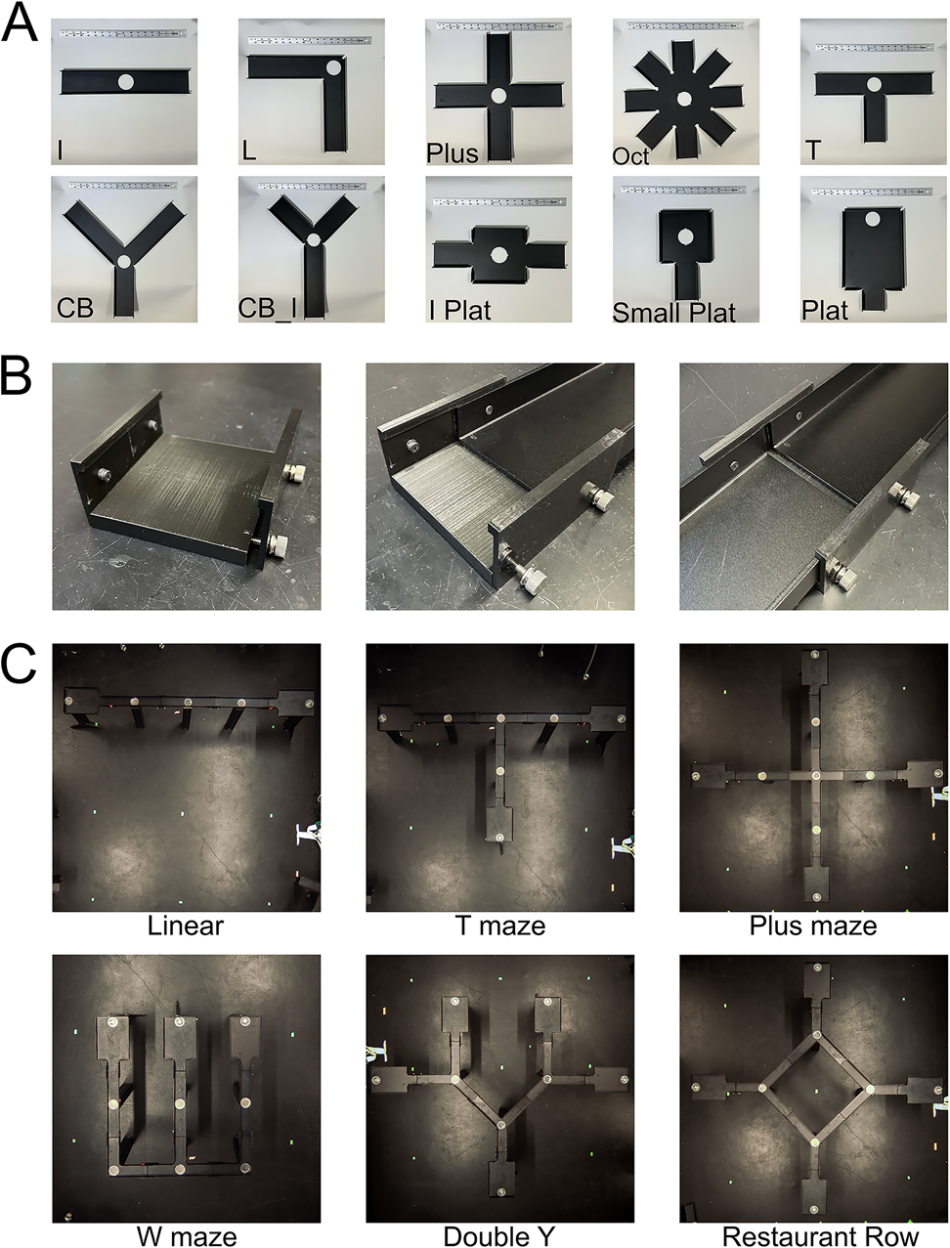
Adaptable maze system. ***A***, Mazes are constructed by combining track pieces into the desired shapes. Pictured are the 10 most common track piece shapes. Depicted ruler is 18 in. ***B***, Track joints support and join track pieces. ***C***, Six example mazes, including common mazes from the neurophysiology literature. Only track pieces are shown; see Extended Data Figure 1-1 for fully assembled complex mazes. CB, corner branch; Oct, octagon; Plat, platform.

Animals move on an interlocking system of custom anodized aluminum track pieces to form the maze environment. Track pieces are all 3″ (7.6 cm) wide with 7/8 in (2.2 cm) walls and come in variable shapes and lengths primarily designed for an 18 in grid (Fig. 1). Track pieces are connected using custom 3D-printed (Ender3 Pro) polylactic acid plastic track joints with 0.25 in (0.6 cm) gaps between tracks (Fig. 1*B*). Barriers necessitate wider gaps of 3 in. Barrier track joints can be used throughout to maintain grid spacing. Each track piece has a 2.5 in (6.4 cm) hole for insertion of a custom reward well or plug (Fig. 3*A*) manufactured in-house using stereolithography 3D printing (Form2 and Form3 printers with clear resin, Formlabs).

#### Leg assembly

Each individual track piece is supported by its own leg assembly (Fig. 3*F*) using a custom quick-lock system (Fig. 3*B*). Contained within the leg assembly is the necessary electronics for reward wells.

Legs are made from 3 × 3 in T-slotted beam (part 3030, 8020 Inc.) and a custom 3D-printed base plate (Fig. 3*C*). Fifteen-inch-tall legs have been extensively used with rats with few adverse events (e.g., rats attempting to climb down). We have utilized legs as tall as 36 in to further discourage climbing down. Legs of any height can be ordered to suit experimental needs. The base plate is attached to the top of the T-slotted beam to lock in with the connector.

The leg assembly is constructed by inserting a reward well or plug through a track piece and screwing it onto the connector (Fig. 3*D*). The track assembly can then be installed onto the leg using the quick-lock system, which requires insertion of the connector hooks into the base plate, a 10° twist into position, and a press down to lock onto the base plate.

#### Automated reward system

An infrared beam break is integrated into the reward well to detect licks (Figs. 3*C*, 6*E*), and reward delivery tubing is routed through the bottom of the well to deliver liquid reward. Port entry detection, licks, and reward delivery are automated using custom hardware and software connected into the SpikeGadgets environmental control unit (ECU). This setup allows for precise control and automation of custom experimental setups using high-level programming languages such as Python or MATLAB (MathWorks). However, other controllers capable of TTL (e.g., Arduino, Raspberry Pi, NeuraLynx, Digital Lynx, Open Ephys) could easily be used instead of SpikeGadgets.

In almost all of our tasks, we have implemented a lick requirement before a reward is dispensed. With the exception of initial training on a linear track, we require rats lick at a reward well for a certain number of licks before a reward is released. This requirement is a behavioral readout that the rat chose an option, rather than just traveled to an area with no intention of collecting a reward. Furthermore, the requirement protects against random footfalls or tails from triggering a reward. We generally we count beam breaks that last >25 ms but <250 ms as “real licks.” Occasionally we need to adjust these parameters, especially the top end duration, for specific animals. We have provided out the shaping code to train the animals to lick on the Gitlab repository.

The current reward well designs do not have a drainage system. We recommend the above lick requirements to avoid erroneously dispensed rewards. However, there may be situations where rats do not consume all the dispensed rewards. If such a scenario is anticipated, it may be beneficial to track the reward wells that had reward dispensed but a consummatory bout of licking did not follow. This may lock out that well from dispensing more reward on the next visit. A drainage system may be possible but would require the modification of the reward well and likely the reward well PCB. Furthermore, while we have not tested this ourselves, the syringe pumps are capable of withdrawing which could remove unconsumed liquid.

#### Automated barriers

**Figure 2.**
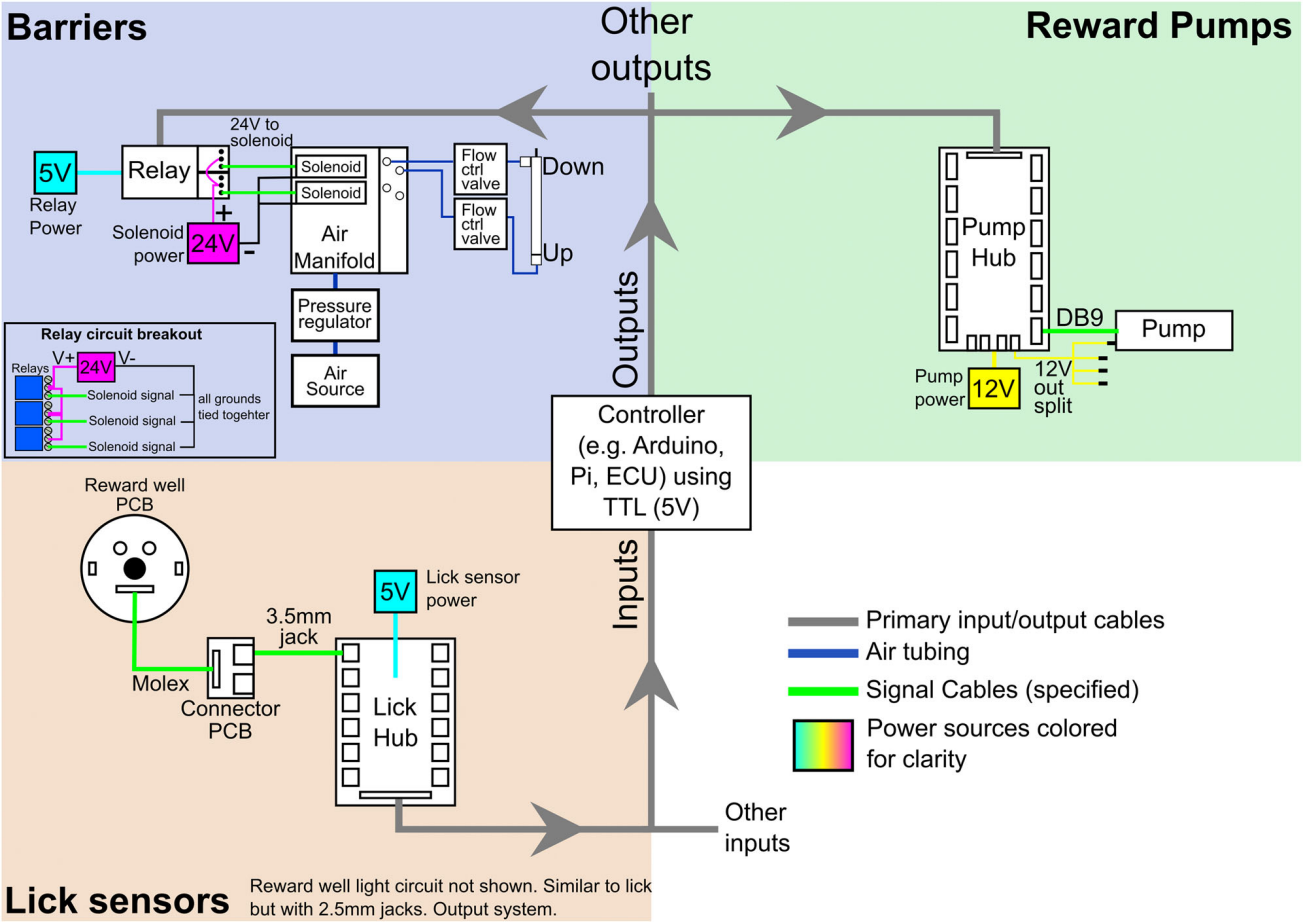
Simple schematic of an example AAM DIO setup. The depicted setup contains reward pumps, lick sensors, and barriers. A similar schematic but with pictures can be seen in Extended Data Figure 2-1.

Automated barriers can be integrated into the maze environment between any two track pieces for within-experiment adaptation of the environment (Fig. 4). Barriers are integrated into the track joint pieces and rise from between the two pieces using a pneumatic cylinder. Air input to the cylinders is controlled via 24 V solenoids connected to a relay board connected to the ECU (Fig. 2). Any microcontroller capable of TTL could control the barriers’ states as well. While pneumatic cylinders are audibly loud, rats habituate to the sound within 1–2 exposure sessions (Movie 1). Pneumatic cylinders avoid electrical interference from alternative methods such as stepper motors (which we tested to great lengths but found them to be unsatisfactory). Each barrier's speed can be fine-tuned via flow control valves. At 12 PSI, we set our flow control valves so that the barriers move both up and down in 0.4 s. However, the air cylinders are rated for 250 PSI, so the barriers’ speed could be made incredibly fast. The flow control valves could also be adjusted to move the barriers very slowly if needed (e.g., <1 cm/s). If the experiment allows, we recommend only moving barriers when rats are at known safe location, such as licking at distant reward wells, to eliminate the possibility of injuring the animal. However, with careful barrier design and care to minimize any gaps in the track pieces, the moving barriers pose little risk to rats’ tails (Movie 1). The barrier system only needs ∼10 PSI to move the barriers and even less if moving one barrier at a time. The barrier air source will ideally be via the building's air source. If unavailable, small commercial compressors can be used. However, these are loud during compression cycles and should be located outside the experimental rooms.

**Movie 1.** Example of implanted rat behavior using the automated barrier system in the van der Meer lab. A male Long–Evans rat was trained to shuttle between the two ends of the linear track for food; later on, a delay zone was added that required the rat to nosepoke for a certain duration in order for the barrier to lower. In this case, the rat was implanted with a 32 tetrode hyperdrive connected to a SpikeGadgets recording system, which can be used seamlessly with the barriers. [View online]

#### Barrier relay circuit

We utilize relays so that the 5 V signals from the controller (1) can control the 24 V solenoids and (2) electrically isolate the solenoids from the controller. The circuit schematic can be seen in Figure 2 and is pictured in Extended Data Figure 4-2. This circuit daisy chains the 24 V power supplier to each relay's common (COM) port, meaning there will be two wires in each COM port except for the last one. You can then use either the normally open (NO) or normally closed (NC) ports to connect the positive lead to the solenoid. Depending on using the NO or NC, the barriers can start in the up or down position when the system is first powered on.

#### Maze assembly times

Initial setup of electronic parts can take a full week, such as soldering PCB components and hooking up connections from the hubs to the ECU/controller. However, once the hubs are hooked up in an experimental room, setting up usable mazes is quick. New users take ∼30 min to learn how to set up a linear track (three straight tracks with small platforms at either end) for the first time. Assembly includes connecting the track pieces and legs, connecting the lick sensor and pump cables, and connecting the two syringes. Experienced users can assemble a linear track in ∼10 min. To assemble an eight-arm radial maze with a barrier on each arm, it takes an experienced person an hour. The increased time is mainly due to all the tubing required for the barriers.

#### Cleaning procedures

Always consult your Institutional Care and Use Committee when developing cleaning protocols. Between each animal, we remove feces and urine and then clean the track pieces with bleach (10% sodium hypochlorite) followed by distilled water. Care is taken to ensure bleach does not get on the reward wells. Reward wells are cleaned of any excess reward with paper towels and water. At the end of each day, we remove the reward dispensing syringes from the maze and clean the tubing. First, the syringe plungers are pulled to remove excess reward. Second, a container is placed at the lower end of the reward well tube. We place the tip of a 60 ml luer lock syringe full of water into the reward well hole. We designed the reward well so that the hole is sealed by the tip of the syringe. Approximately 20 ml of water is then forced through the tubing and collected in the container. If viscous solutions such as high concentrations of sucrose or evaporated milk are used, we recommend cleaning the tubes with pipe cleaners once or twice a month to avoid buildup. While rare with regular cleaning, it 's possible that the reward solution can build up on the IR emitter and/or receiver on the reward well. A stiff bristle brush and water can remove any buildup.

### Experimental methods

All experimental methods were approved by the Brandeis University Institutional Care and Use Committee (#21001, #24001-A) or the Dartmouth College Institutional Care and Use Committee (#00002154). All experiments were conducted under the guidelines of the US National Institutes of Health. Data from one adult male TH-Cre rat (445 g, 5 months old; Witten et al., 2011) is presented here; however, dozens of rats have carried out experiments on the AAM. The animal participated in a previous experiment (Ding et al., 2025) before participating in the radial arm maze associative memory task. Detailed methodological procedures including surgery are described in Ding et al. (2025). Briefly, the animal was singly housed due to neural implantation and given *ad libitum* access to food and water on a 12/12 h light/dark cycle. Lights were on from 7 A.M. to 7 P.M. Prior to training, the rat was food restricted to no <85% of his free feed weight. The rat was habituated to the experimenters and evaporated milk before being trained to shuttle back and forth on a linear track. Once readily shuttling, the rat underwent stereotaxic surgery and was injected with AAV5-EF1a-DIO-hChR2(E123T/T159C)-mCherry into the VTA and implanted with a custom hyperdrive consisting of tetrodes bilaterally targeting dorsal CA1 and prefrontal cortex and optrodes (optical fiber with tetrodes) to the VTA. The rat was given 3 weeks to recover while the virus expressed, tetrodes were lowered to their target areas, and it was retrained on the linear track. The rat was then trained on a W maze-based rule switching task.

After completion of the W maze rule switching paradigm, the rat was trained and tested on a sequence running task where each arm of the radial arm maze (Fig. 6*A*) represented an item in the sequence. Utilizing the barriers, on a given trial, the rat started at a Home arm and was presented two choice arms to choose from (Fig. 5). Given the presented arms, the rat had to run the correct sequence from one to another. If correct, the rat received a reward at the first arm and the Home arm was blocked (“Barr 1”). The rat would then proceed to the second arm to get a second reward. After the second reward was delivered, the barrier to the first arm was raised (“Barr 2”), and the Home barrier was lowered (“Barr 1”) to start another trial.

The session was recorded with an overhead camera (Mako G-158C, Allied Vision) recording at 30 fps. The speed of the rat was calculated by tracking an LED on the headstage (SpikeGadgets) of the animal in DeepLabCut (Nath et al., 2019) and calculating its instantaneous velocity and smoothing with a centered moving average of seven frames. Hippocampal local field potential data were acquired via a SpikeGadgets MCU and exported with a low-pass filter with a cutoff of 500 Hz. Theta was filtered at 8–12 Hz using a third-order Butterworth filter. Spiking data were obtained using MountainSort (Chung et al., 2017) and manually curated with Plexon OfflineSorter (Plexon) primarily using principal components and peak-valley distance to visually inspect cluster quality.

## Results

We designed a maze system using modular track pieces (Fig. 1*A*) that enables the creation of a wide array of two-dimensional track environments (Fig. 1*C*; see Extended Data Fig. 1-1 for complex mazes). All the necessary files and materials can be found on the Gitlab repository (https://gitlab.com/jadhavlab/AdaptAMaze/-/tree/main?ref_type=heads). We chose to make the track pieces out of aluminum for a variety of reasons. Using metal ensures the track pieces are strong, long-lasting, nonporous, and easy to clean and minimize static buildup on the animals. We have noted during low-humidity periods that static discharge can occur. Increasing the room's humidity ameliorates the issue. We specifically selected aluminum as it is an inexpensive sheet metal that is lightweight and corrosion resistant (comparative to stainless steel). It 's a soft metal that allows for modifications if needed with simple tools (e.g., handheld drill or hacksaw). Furthermore, aluminum can be anodized to a matte black surface, as we have done, to minimize light reflections to improve animal tracking. Each track piece is supported by a leg, and track pieces are joined using custom 3D-printed track joints (Fig. 1*B*). Track pieces are designed to meet at locations on an 18 inch grid (except for the platform pieces), thereby allowing any combination of pieces to be used to create the maze of choice. The system is scalable, as mazes can be expanded by simply adding another piece. It is also flexible, as a maze can be adapted by replacing one or more pieces by simply disconnecting the joint connections and placing alternative track pieces. Due to the standardized design and componentry, maze environments can be easily recreated both in and across labs, greatly enhancing repeatability.

10.1523/ENEURO.0138-25.2025.f1-1 Figure 1-1 **Complex maze setups.** Members of the lab have implemented a variety of complex mazes in addition to common configurations (e.g. linear, W, T; **Figure 1C**). **A)** Classic radial arm maze used for a transitive inference task. Reward wells were located at the end of each arm. Barriers were placed at the start of each arm to control access to arms. Various materials were attached to the track pieces of each arm for unique local cues. **B)** Large (90” x 108”) 5x5 grid maze with a home arm (bottom left **Bi & Bii**) used for a memory schema paradigm. Reward wells were on each “node” of the grid. **C)** Large, complex maze with rewards available at a home location and four outer arms. Multiple routes could be taken from home to the outer arms. Available routes were manipulated via barriers and/or moving the home arm between sessions to test spatial memory. Download Figure 1-1, TIF file.

**Figure 3.**
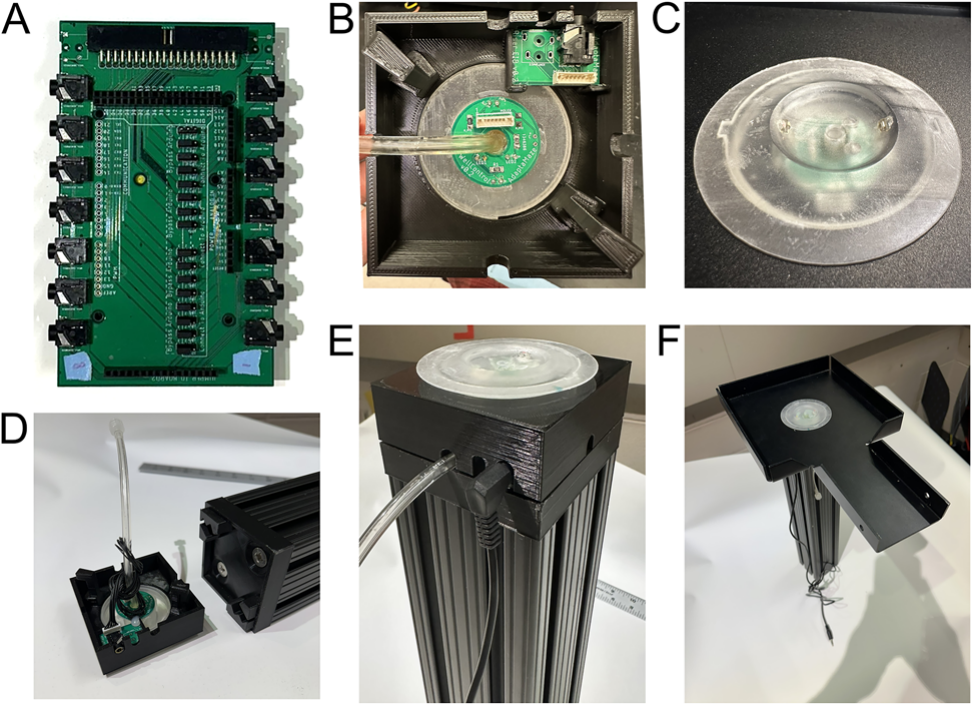
Integrated automated reward wells. ***A***, Reward well detection command PCB to route lick sensor cables from reward wells (shown in ***B***) to ECU/controller and handle power to LEDs. ***B***, Underside of constructed reward well and leg connector. No wires shown. ***C***, Example reward well with infrared (IR) emitter and phototransistor aligned across the liquid spout. ***D***, Assembled reward well in connector (as in ***B***) and leg with baseplate. ***E***, Constructed “leg stack” with no track piece. ***F***, Fully assembled modular track piece. Detailed parts breakouts for the reward well, track joint, and reward pumps are shown in Extended Data Figures 3-1 and 3-2.

All aspects of the AAM system can be automated including lick detection, reward dispensing, and barrier movements (Extended Data Fig. 2; Fig. 2-1). The maze system features automated reward well lick detection and liquid reward distribution (Fig. 3; Extended Data Figs. 3-1, 3-2). A custom PCB (Fig. 3*A*) routes 5 V signals from detected licks from each reward well to the digital input/output (DIO) controller (e.g., ECU from SpikeGadgets, Arduino, Raspberry pi, etc.). Integrated into each reward well is an infrared beam break circuit, two visible signaling LEDs, and tubing for liquid reward delivery (Fig. 3*B*,*C*). Each track piece is held in place by either a reward well or a flat “plug” that does not have a well or hole (data not shown). A reward well or plug is screwed into the leg connector (Fig. 3*D*) attached to the supporting leg baseplate (Fig. 3*E*). These components are tucked into the leg assembly upon construction (Fig. 3*E*,*F*), protecting componentry from curious rodents and allowing for quick connections and easy cleaning. Liquid rewards were chosen to minimize chewing artifacts common to solid food rewards (Mondragón-González and Burguière, 2017; Fabietti et al., 2020). Custom software controls the SpikeGadgets ECU for reward programs. Further control over reward delivery can be programmed to avoid. For example, we program a lick requirement so that reward is dispensed only after a rat licks a certain number of times at a well. This requirement serves as a behavioral readout of the rats choice and prevents unwanted events from dispensing milk, such as the rat standing on the well. A similar PCB controls the visible LEDs in the reward wells, and a third PCB controls the pumps to deliver liquid rewards (all PCBs, including circuit diagrams: https://gitlab.com/jadhavlab/AdaptAMaze/-/tree/main?ref_type=heads). If desired, an Arduino can interface directly to the PCBs to log licks or deliver rewards.

10.1523/ENEURO.0138-25.2025.f2-1 Figure 2-1 **Picture-based schematic of AAM DIO system.** Same as **Figure 2** using SpikeGadget’s ECU as the controller. Download Figure 2-1, TIF file.

10.1523/ENEURO.0138-25.2025.f3-1 Figure 3-1 **Reward well and track joint parts.** Part breakouts for **A)** reward well & connector, **B)** track joint, and **C)** lick detection hub seen in **Figure 3**. Part IDs reference the parts list. Part ID colors correspond to the primary system they are associated with and are consistent with the Parts List’s tab colors. Red = track parts, green = reward wells, blue = reward delivery, grey = DIO connections. Download Figure 3-1, TIF file.

10.1523/ENEURO.0138-25.2025.f3-2 Figure 3-2 **Reward pump parts.** Part breakouts for **A)** Reward Pump hub, **B)** underside of reward pump hub, and **C)** syringe pump for Reward system (**Figure 3**). Part IDs reference the parts list. Part ID colors correspond to the primary system they are associated with and are consistent with the Parts List’s tab colors. Red = track parts, green = reward wells, blue = reward delivery, grey = DIO connections. Download Figure 3-2, TIF file.

**Figure 4.**
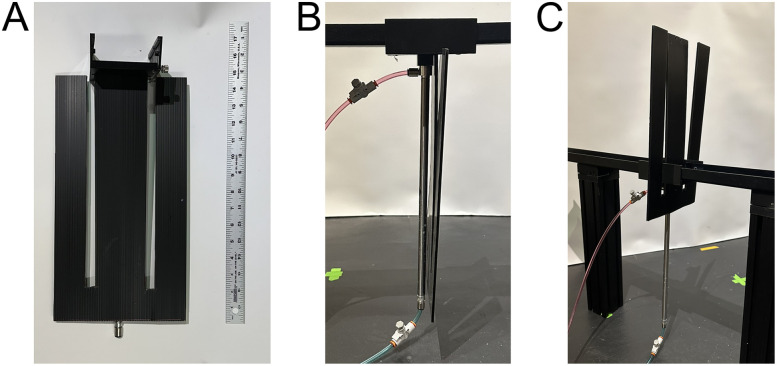
Automated barriers. ***A***, The front view of barrier assembly with corrugated plastic barrier attached to 3D-printed track joint. ***B***, The side view of barrier assembly in the down position between two track pieces. Flow regulators can be seen on tubing prior to the tubing's connection to the piston. ***C***, Barrier in the up position. Extended Data Figure 4-1 shows detailed part breakouts for barrier system including pneumatic control.

To achieve environmental flexibility within a recording session, the maze system includes the ability to place automated barriers between any two track pieces (Fig. 4, Extended Data Fig. 4-1). Barriers are pneumatically driven to avoid electrical noise commonly generated from electric motors. Like the reward system, barrier position is controlled through digital inputs from the ECU to a simple relay interfacing with solenoids to direct airflow to the appropriate barrier. Barrier speed can be individually and directionally (i.e., up and down) controlled via flow regulators (Fig. 4*B*,*C*). Barriers can be made to a variety of heights but are limited by piston length (McMaster-Carr supplies these pistons with stroke lengths up to 36 in) and maze leg height. Commonly, barriers are constructed from corrugated plastic which is easy to work with, inexpensive, and cleanable. Clear Plexiglas barriers have been used to block routes while maintaining visual cues. However, any material could be used as a barrier with sufficient air pressure. Overall, due to the modular design of the AAM, certain components such as the reward wells or lick sensors could be adapted by other labs for their mazes (e.g., the automated barriers were used in two different labs; Movie 1).

10.1523/ENEURO.0138-25.2025.f4-1 Figure 4-1 **Automated Barrier parts.** Part breakouts for **A)** barrier with track joint, **B)** barrier fastener, **C)** relay for barriers, and **D)** air manifold for Automated barrier system (**Figure 4**). Part IDs reference the parts list. For relay board (PB1) in **C**, do not use Vcc for power as it will pull power for the relays from your controller. Use an external power source (PB5) to JDVcc. Part ID colors correspond to the primary system they are associated with and are consistent with the Parts List’s tab colors. Red = track parts, green = reward wells, blue = reward delivery, grey = DIO connections. Low opacity indicates part is not visible in picture from this viewing angle. Download Figure 4-1, TIF file.

**Figure 5.**
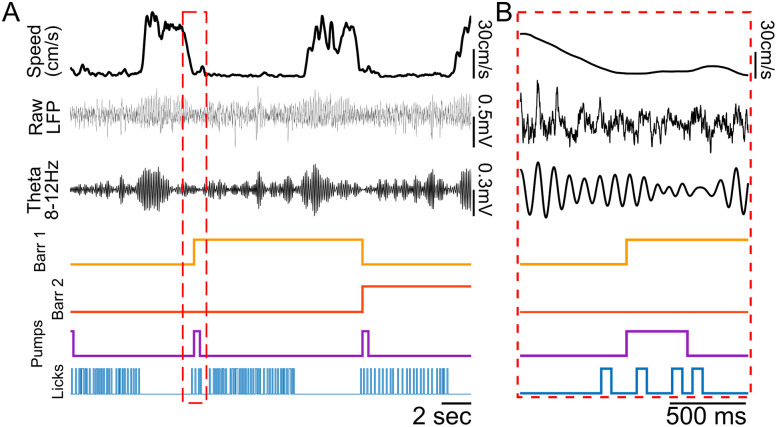
Example trial. ***A***, Traces are from one trial of a rat running a memory sequence task on a radial arm maze. Top, Rat's running speed as he consumed rewards and ran between arms. Raw LFP recorded from the HPC (dorsal CA1) and the LFP filtered for the theta rhythm (8–12 Hz). Barr 1 and 2 depict control signals for two different barriers as they are automatically controlled depending on the arm the animal is on. “High” signal indicates a raised barrier. Three reward pump signals were concatenated to one trace (“Pumps,” purple). A reward is dispensed while signals are high. The final trace shows putative licks detected from the integrated reward well IR beams at three different reward wells (“Licks,” blue). “High” indicates the beam was broken. ***B***, Zoomed in portion of ***A*** (red dashed square) showing no artifact in the neural traces when both the barrier (yellow) and reward pump (purple) are turned on. Also depicted is our lick requirement where reward was only dispensed once a rat initiated licking to indicate their choice.

**Figure 6.**
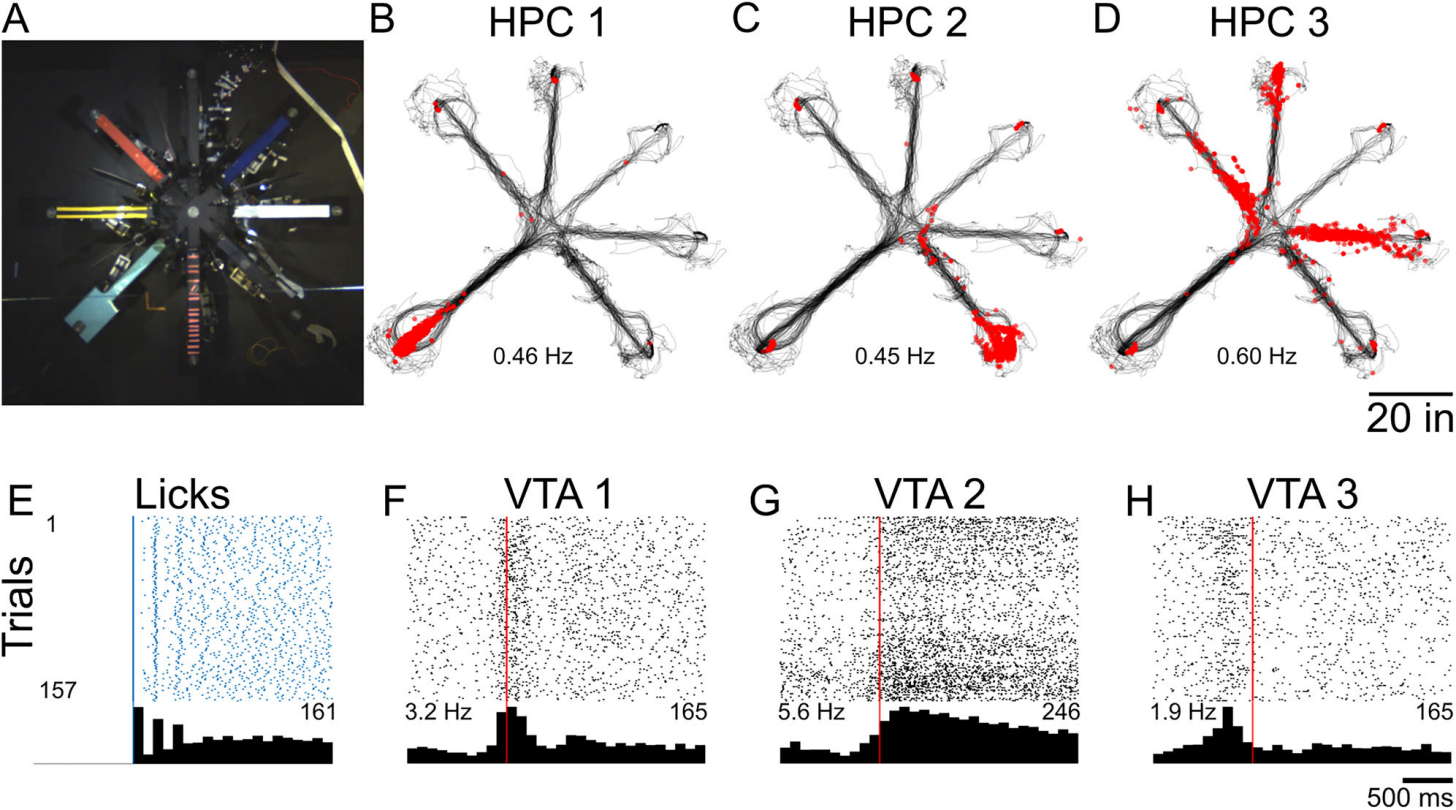
Example neuronal activity on an AAM task. ***A***, Eight-arm radial arm maze used to test sequence memory in rats. ***B–D***, Example plots of three HPC neurons from one recording session on the radial arm maze. Black lines depict the rat's position on the maze. Each red dot is the location where an action potential was fired. Mean firing rates for the recording sessions shown below each plot. Neurons shown in ***B*** and ***C*** are spatially selective, firing to the end platforms of an arm. The cell in ***D*** shows selective spatial firing to multiple arms. ***E***, The raster plot and perievent time histogram (PETH) centered on the first lick of correct trials from the same recording session across all reward wells. Inset right is peak lick count. ***F–H***, Rasters and PETHs of three example VTA neurons around reward consumption. Red vertical line indicates first lick. Mean firing rates for the recording session are inset on the left, while max spike count on the histogram is inset right. A variety of responses were observed including phasic response at reward delivery (***F***), tonic response during licking (***G***), and preemptive firing before lick onset (***H***).

The AAM has been extensively tested over the past 5 years in our lab. We have used the AAM in a variety of configurations including linear tracks, W mazes, eight-arm radial mazes, and other complex configurations (Extended Data Fig. 1-1). The mazes have been tested exclusively with *ad libitum* behaving rats (male and female Long–Evans aged 3–13 months). Researchers utilizing mice may want to modify the track pieces to be smaller. Some rats were implanted with hyperdrives, optical fibers, or both to a variety of brain regions including the hippocampus (HPC), prefrontal cortex, and ventral tegmental area (VTA). Figure 5 depicts example data from one trial from one animal running a radial arm maze (Fig. 6*A*) that had automated barriers to control arm access (see Movie 2 for an example trial). We have utilized the AAM to investigate a wide range of system neuroscience questions including the hippocampal representations of complex sequences (Fig. 6*B,D*) and VTA neuronal responses around reward delivery over learning (Fig. 6*E*,*H*). We look forward to the limitless possibilities other researchers come up with using and adapting the AAM system.

**Movie 2.** Example trial of rat running on an AAM eight-arm radial maze, similar to those shown in Figures 5 and 6, in the Jadhav lab. Rat was implanted with a 64-tetrode drive and tethered while running. Neural data were acquired with a SpikeGadgets system that also controlled the automation of the maze. The rat had no issues with the barrier sounds nor the tether getting caught on any part of the maze. [View online]

## Discussion

Here we have detailed a novel modular maze system for behavior and systems neuroscience research in rodents. The design integrates reward ports into the track pieces and includes automated lick detection and liquid reward distribution. Automated barriers can be included between any two track pieces, furthering flexibility of the design. Together, our open-source design comprises a simplified system capable of replicating standard maze configurations as well as novel experimental designs.

Notably, this design allows for the construction of stereotyped and clearly documented maze designs within and across labs, increasing repeatability within an experiment and the ability for labs to replicate the same experimental setups. For example, classic spatial navigation and working memory assays on environments such as “T,” “Plus,” and “W” mazes can be constructed with this system to exact specifications and with shared, standardized control code. These same setups can then be exactly replicated without variability in implementation due to varied hardware (e.g., material, port type) or behavioral task control code. Furthermore, the automation of reward delivery and barriers could potentially reduce variability in experiments that previously had experimenters manually releasing rewards or adjusting barriers.

Another benefit of a modular system is the flexibility to reuse lab spaces and adapt maze designs with ease. A common maze setup is a single-piece or fixed design. For another researcher to then use that same experimental space with a different maze design, the first maze must be relocated. Obviously, this limits the feasible number of actual mazes used in a lab. In addition, if two or more mazes want to be used for the same experiment session, it may be difficult to make the transition in a timely manner. With our system, this can be addressed quickly and easily with the repositioning or exchanging of track pieces, facilitating both minor space manipulation experiments in minutes or complete track redesigns in tens of minutes for a single experimenter. The modular aspect also facilitates simple scaling of mazes by adding segments to existing track pieces, something impossible with fixed or single-piece designs.

Perhaps the most exciting benefit of this system is the reduced financial, engineering, and setup costs of experiments. Spurring the AAM was the inordinate costs many companies charge for prebuilt automated mazes that range from thousands to tens of thousands of dollars and often require additional integrations (e.g., lick sensors) at additional cost. Furthermore, many commercial mazes use proprietary software that can limit integration into other systems (e.g., electrophysiology rigs) and impede reproducibility. In March 2025, we were quoted $27,744 USD for an automated eight-arm rat maze from a well known maze company. In contrast, an AAM system for an eight-arm radial maze purchasing 10 pieces of straights (I) and platforms (Plat_small) and two eight-arm intersections (Oct) would cost ∼$9,500 (price assumes the lab has 3D printers). Specifically, the track pieces would cost ∼$2,000, legs $750, barriers $1,800, reward delivery $4,300, and smaller parts making up the remainder (see Parts List for detailed pricing). Substantial savings (∼$4,000) are possible by using peristaltic pumps ($200) rather than syringe pumps. Importantly, the same track pieces could be reused to construct multiple linear tracks, two four-arm plus mazes, and three T/Y mazes. The AAM system also reduces new maze prototyping times by months or even years. Our open-source designs mean researchers could adapt our existing track pieces, reward wells, barriers, and circuits to meet their needs. For example, the lick sensors could be adapted to nose poke sensors, and the barrier system could be made into an air puff system. Because of the minimal cost of any particular configuration, many configurations can be quickly piloted to find the preferred environmental design.

This maze system is compatible with behavioral recording as well as neurophysiology recording techniques, including in vivo electrophysiology and one-photon calcium imaging. System components are entirely below the track surface, clearing the space above the track for unimpeded visual recording or physical tethering. In particular, this design will enable continuous neural monitoring during behavioral tasks in multiple maze environments.

The AAM does however have some limitations. Simple translations or rotations of the maze in the room are difficult with the current design, as the connections between track segments are weak compared with the weight of the legs. One possible solution is to connect the legs with additional T-slotted beams to add strength, but this will trade off with the amount of time to adapt the system. Two-dimensional mazes are very stable on their own due to the weight of the legs and the track pieces bracing one another. However, linear tracks can be unstable in the sense that a forceful bump could knock it over. In tight quarters where the risk of bumping a linear track may be high, using T-slot accessories to widen the leg base may be helpful. Another concern is the extent of design flexibility. Our maze system is currently laid out on a grid system for ultimate flexibility. As is, the grid does limit the potential maze designs for certain radial configurations. However, users can easily modify or create new track pieces or customize components to add functionality to the system. The only compatibility requirements are to interface with either our track joint and reward well designs or the basic sheet metal track pieces. Finally, the current track pieces are incapable of three-dimensional maze designs, so unique mazes (Wilson et al., 2015; Grieves et al., 2020) will still be required to explore three-dimensional navigation. Three-dimensional “surfaces” could be possible with varied leg heights and bends in the track pieces.

We have encountered some common wear and tear items that may be useful to point out. Some of our mazes utilize the barriers of every trial, resulting in hundreds of up–down cycles per day. Over time, the barrier material (if using corrugated plastic) can dislodge from the air cylinder. Daily checks to examine their stability are helpful. Even so, replacements are only needed every year or so. Moreover, a subset of rats may become frustrated when not receiving a reward at a well and will attempt to chew it. We considered this in the reward well design by utilizing smooth surfaces. However, given enough time, their teeth can scratch the plastic enough it 's worth replacing to ensure cleanability.

There are many alternative possibilities with different relative costs and benefits. Single-piece mazes made from materials such as wood are quick and cheap to construct but are permanent in their design and occupy considerable experimental space when not in use. Furthermore, some institutions do not allow wooden mazes for animal use due to being porous and hard to clean. Complex, unique mazes can be perfectly catered to a single experiment but often have immense engineering setup costs and permanent designs. Another maze alternative is a virtual reality (VR) setup. The utility of VR setups as compared with our design is similar to other real-world mazes (Minderer et al., 2016; Chen et al., 2018). VR offers the ability to precisely control sensory variables and even decouple typically coupled variables such as movement speed and visual flow, but that same decoupling can also be a disadvantage and creates an unnatural situation that may not be desired for the study. VR setups can also be more amenable to head-fixed recording techniques, though simple mazes are now possible on head-fixed setups (Kislin et al., 2014). Finally, an impressive modular track system was recently published by Hoshino et al. (2020). Their system boasts many of the features of our system but with a few important distinctions. Their reward system is for solid food as opposed to liquid, and their barrier system is not automated. Hoshino et al. also use a grid system, but it is implemented on the floor, limiting their ability for configurations to 45 and 90° segments. They have developed a beam break system that is decoupled from reward locations, allowing for more flexible behavioral control than currently available with our AAM system. Many of these variations could be added to our system due to its fundamental simplicity, but they are not currently integrated.

Mazes remain a ubiquitous tool in rodent behavior and systems neuroscience (Wijnen et al., 2024). We have designed a modular maze system to enable flexible maze designs and rapid experiment prototyping and development. Adaptation of this standardized system will also allow for improvements in repeatability and replication. Overall, the authors believe this maze system adds an important tool for researchers and will facilitate and expedite novel behavior and systems neuroscience discoveries.

## Synthesis

Reviewing Editor: Mark Laubach, American University Decisions are customarily a result of the Reviewing Editor and the peer reviewers coming together and discussing their recommendations until a consensus is reached. When revisions are invited, a fact-based synthesis statement explaining their decision and outlining what is needed to prepare a revision will be listed below. The following reviewer(s) agreed to reveal their identity: Jai Yu, Lisa Genzel.

Thank you for sending your paper to eNeuro! It was reviewed by two experts. Their comments are below. Please revise your paper to address all points that were raised.

Reviewer 1

This is a very useful system for constructing mazes for the spatial learning community. It provides a modular design that simplifies creating different geometric designs and prototyping behaviors. The manuscript is straightforward and clear to understand.

These points could be elaborated on.

1. Aluminum is chosen for the maze sections. Were there other reasons in addition to longevity for this choice? For example, is metal better for electrophysiology to reduce static build up and discharge? Do you find static discharge issues? From a manufacturing perspective sheet metal work still requires specialized workshops or outsourcing. Some perspective on the choice would be great.

2. For the reward delivery system, the placement on the floor of the maze allows for flexibility of reward locations. Is there a drainage system in case a reward is not claimed? Otherwise, the next reward may double the reward size at the same location. How do you deal with this scenario?

3. Given the reward location is on the floor, is there a concern that licks and footsteps may need specific algorithms to distinguish? What does actual data look like for these distinct events? I imagine these will look different, but it would be good to discuss how this may affect behavior analyses.

4. How is the maze stabilized? Are the vertical support legs secured to the floor with screws or other methods? You don't want the maze to wobble.

5. The cost savings is great to know. A mention of the a per piece cost would be useful since this is a modular design. For ~$8000 USD, how many pieces of the track do you get?

6. Given the lab has used this system for a few years, what kind of wear and tear do you find for this system? Are there common issues and solutions under real life conditions?

Minor comments

1. Fig 4. It would be great to put a vertical scale on the LFP traces.

2. Fig. 5. A vertical scale indicating the number of trials, as well as the mean rate for the PETH plot would be useful.

Reviewer 2

This article describes an open-source, low-cost, and automated maze for rats that can be employed in various behavioral paradigms. It is a valuable contribution, particularly for laboratories lacking engineering resources, and it supports efforts to standardize behavioral experimentation. The associated GitHub repository is accessible and well-organized. However, there are several major and minor issues that need to be addressed:

The term reproducibility is used repeatedly throughout the manuscript, yet no empirical data are provided to support this claim. Demonstrating that different behavioral paradigms can be implemented across laboratories does not constitute reproducibility per se. To support this claim, it would be necessary to present the same behavioral task conducted in at least two separate laboratories, showing comparable progression of behavioral performance in the animals. If such evidence is not available, the authors should clarify which aspects of the maze setup are reproducible.

EDITOR'S NOTE: Revisions to the text might be the most direct way to address this concern. However, if data from other labs is available, please include it.

The manuscript also states that the system reduces variability across experimenters, but this claim is not substantiated. It remains unclear how this reduction would occur without a standardized, detailed protocol for each behavioral task.

EDITOR'S NOTE: Again, revisions to the text might be the most direct way to address this concern.

Several technical and practical details are missing, which would improve the utility of the manuscript. These include the minimum and maximum speeds of the barrier movements, the recommended cleaning procedures for the maze, and a detailed cost breakdown of the radial arm maze setup.

EDITOR'S NOTE: Please add the listed details to your paper.

The reported maze assembly time in the methods section appears incomplete, as it does not account for steps such as connecting inputs/outputs.

EDITOR'S NOTE: Please include an estimate of the final setup time, including how long it takes to make these connections.

The figures throughout the manuscript require substantial improvement in terms of design. Specific issues include:

Font size: There is inconsistency in font sizes across figures, with some text too large and others too small to be legible.

Missing elements: A clear image of the maze legs is missing.

Figure 1: The scale of the ruler should be specified.

Figures 2 and Suppl. Fig. 1: Images differ in size; they should be aligned within a uniform grid layout.

Figure 5: The title refers to "AAM tasks," but only a single task is shown.

Supplementary Figure 2: The blue squares are undefined, and the text in the barrier and reward well sections is too small.

Supplementary Figure 3: This figure is helpful in understanding the system, but it requires clearer labeling and consistent font size and positioning. Additionally, the "Power sources colored for clarity" symbol is not applicable here.

Supplementary Figures 4 and 5: Color coding should be explicitly explained, and the layout requires better organization.

Finally, several abbreviations are undefined upon first use, including PCB, IR, and DIO.

In the sentence "VR also enables head-fixed recording techniques," the use of "enables" may not be the most appropriate choice. Consider whether "requires" or another term might better reflect the intended meaning.
