# Supplementary figures and images for: Adapt-A-Maze: An Open-Source Adaptable and Automated Rodent Behavior Maze System
Source: eNeuro. 2025 Jun 17;12(7):ENEURO.0138-25.2025. doi: 10.1523/ENEURO.0138-25.2025 (PMC12243947; doi:10.1523/ENEURO.0138-25.2025)

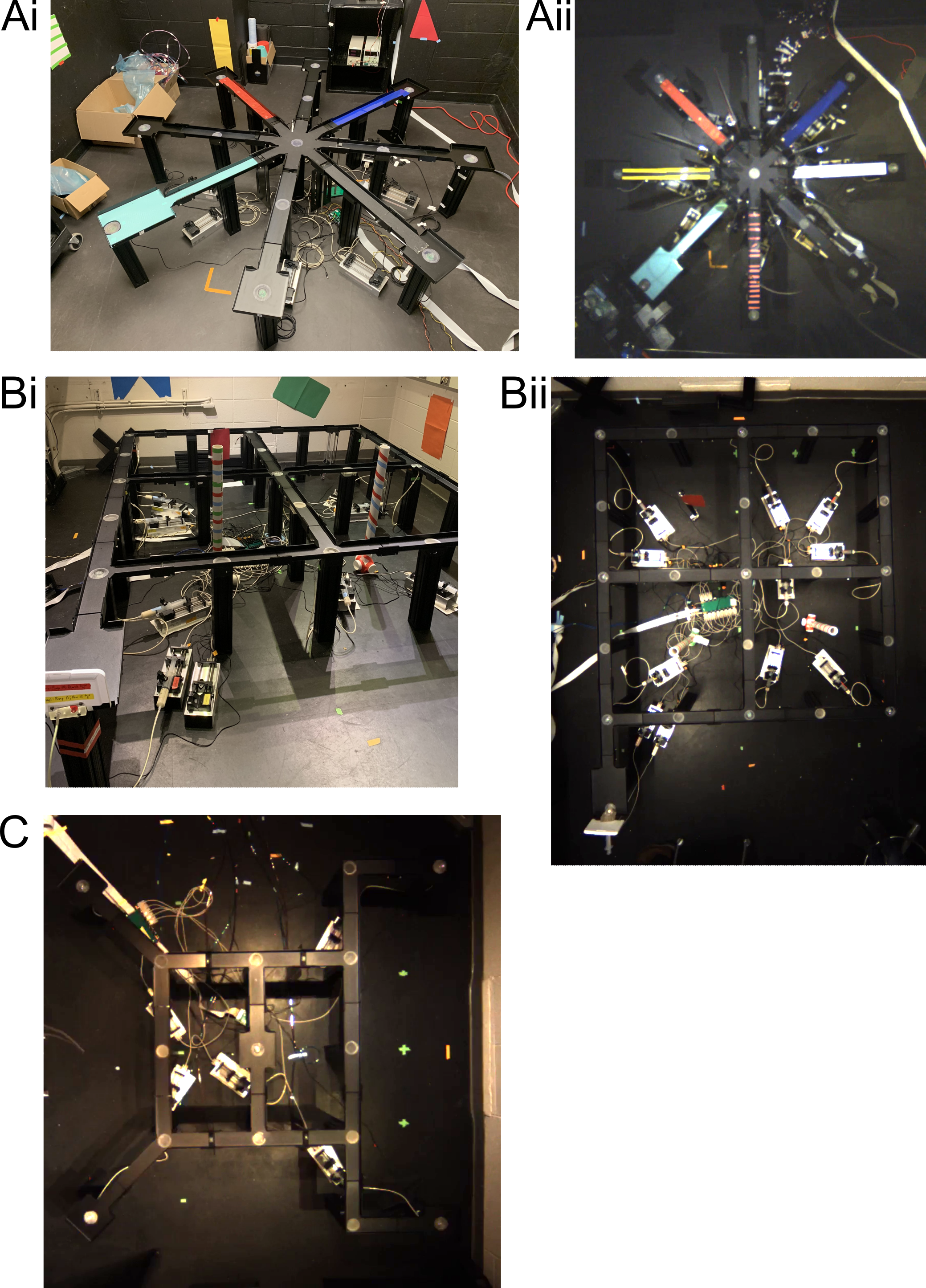

Supplement: Figure 1-1 — Complex maze setups. Members of the lab have implemented a variety of complex mazes in addition to common configurations (e.g. linear, W, T; Figure 1C). A) Classic radial arm maze used for a transitive inference task. Reward wells were located at the end of each arm. Barriers were placed at the start of each arm to control access to arms. Various materials were attached to the track pieces of each arm for unique local cues. B) Large (90” x 108”) 5x5 grid maze with a home arm (bottom left Bi & Bii) used for a memory schema paradigm. Reward wells were on each “node” of the grid. C) Large, complex maze with rewards available at a home location and four outer arms. Multiple routes could be taken from home to the outer arms. Available routes were manipulated via barriers and/or moving the home arm between sessions to test spatial memory. Download Figure 1-1, TIF file. [file eneuro-12-ENEURO.0138-25.2025-s002.tif]

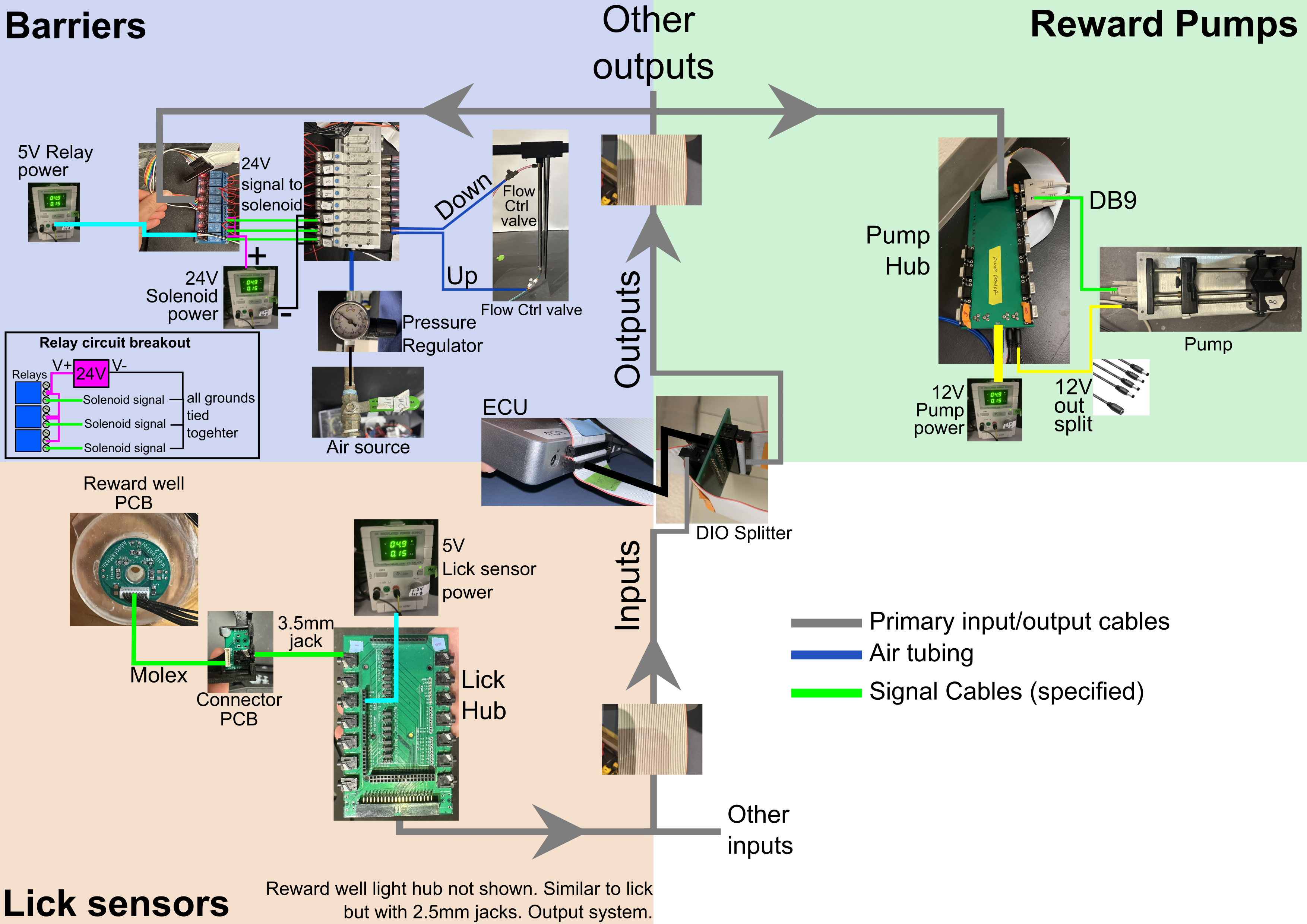

Supplement: Figure 2-1 — Picture-based schematic of AAM DIO system. Same as Figure 2 using SpikeGadget’s ECU as the controller. Download Figure 2-1, TIF file. [file eneuro-12-ENEURO.0138-25.2025-s003.tif]

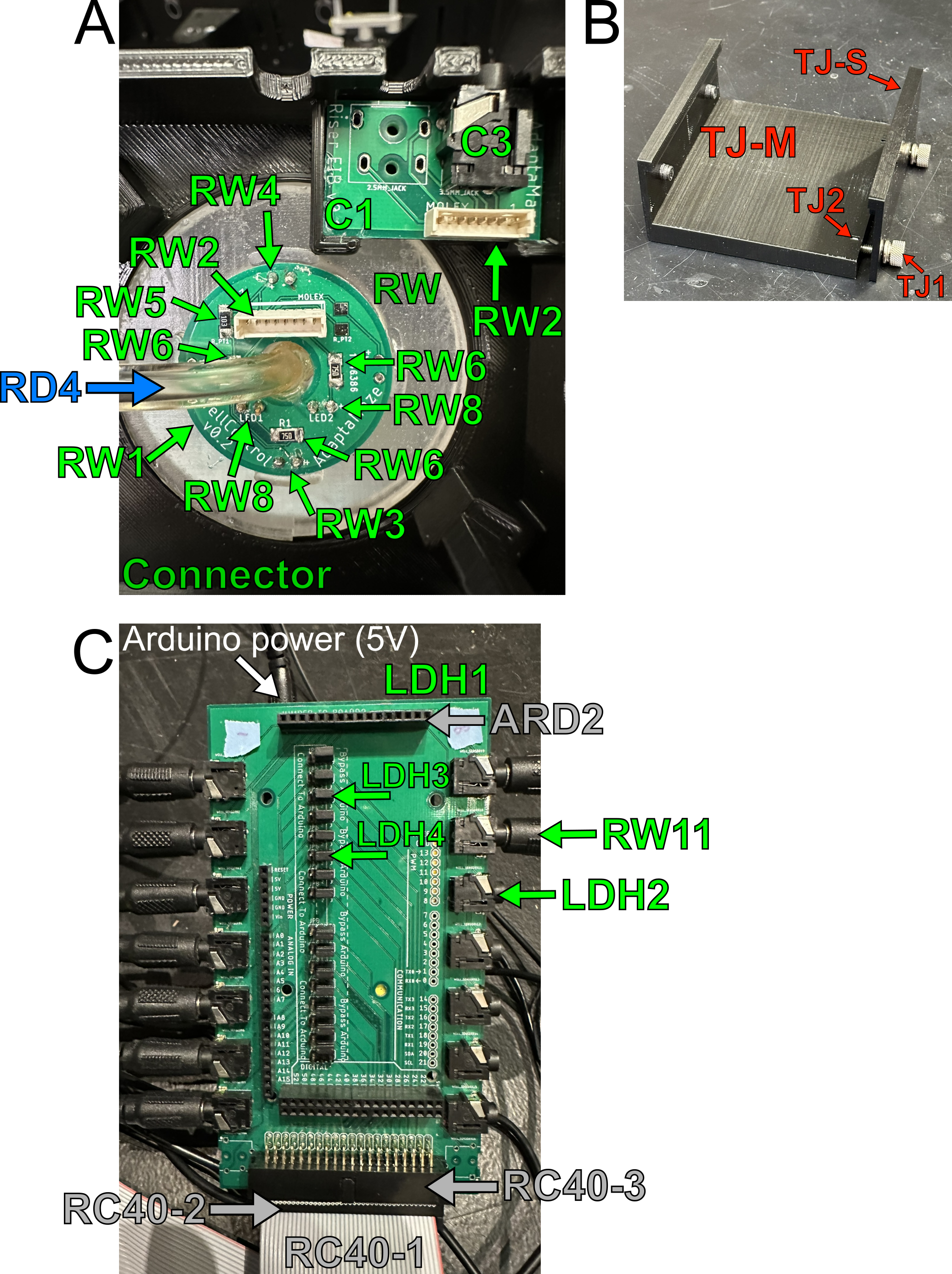

Supplement: Figure 3-1 — Reward well and track joint parts. Part breakouts for A) reward well & connector, B) track joint, and C) lick detection hub seen in Figure 3. Part IDs reference the parts list. Part ID colors correspond to the primary system they are associated with and are consistent with the Parts List’s tab colors. Red = track parts, green = reward wells, blue = reward delivery, grey = DIO connections. Download Figure 3-1, TIF file. [file eneuro-12-ENEURO.0138-25.2025-s004.tif]

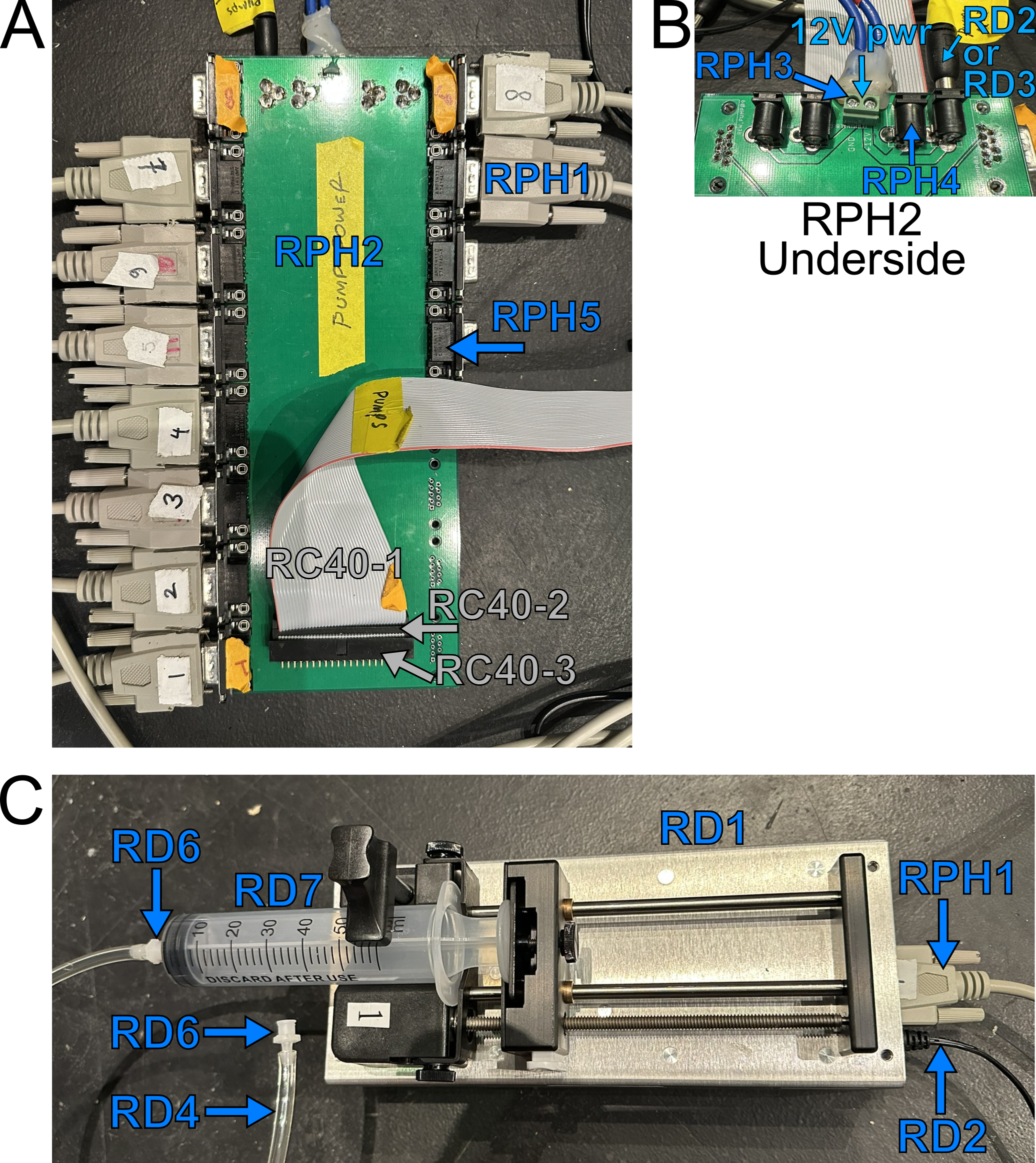

Supplement: Figure 3-2 — Reward pump parts. Part breakouts for A) Reward Pump hub, B) underside of reward pump hub, and C) syringe pump for Reward system (Figure 3). Part IDs reference the parts list. Part ID colors correspond to the primary system they are associated with and are consistent with the Parts List’s tab colors. Red = track parts, green = reward wells, blue = reward delivery, grey = DIO connections. Download Figure 3-2, TIF file. [file eneuro-12-ENEURO.0138-25.2025-s005.tif]

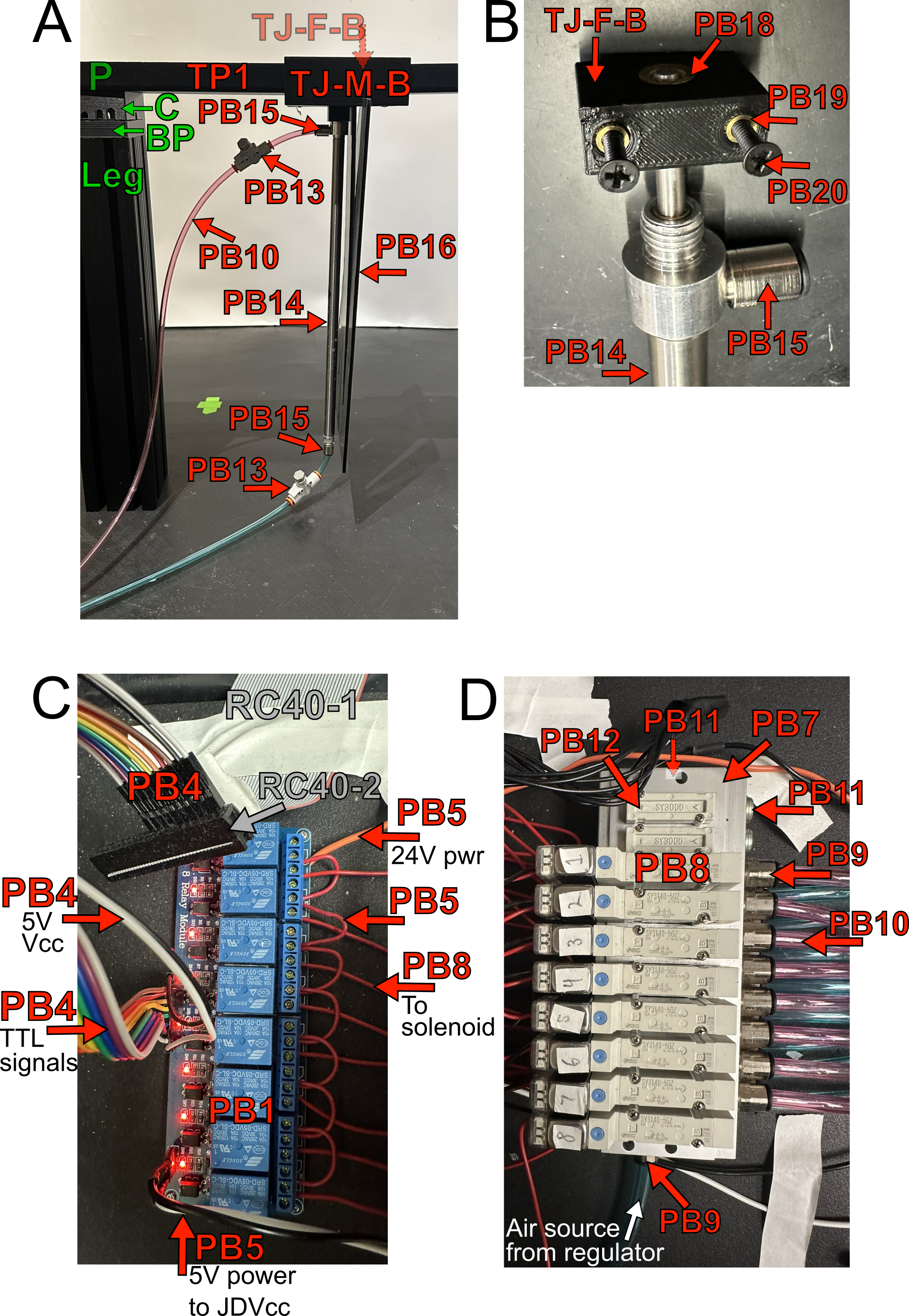

Supplement: Figure 4-1 — Automated Barrier parts. Part breakouts for A) barrier with track joint, B) barrier fastener, C) relay for barriers, and D) air manifold for Automated barrier system (Figure 4). Part IDs reference the parts list. For relay board (PB1) in C, do not use Vcc for power as it will pull power for the relays from your controller. Use an external power source (PB5) to JDVcc. Part ID colors correspond to the primary system they are associated with and are consistent with the Parts List’s tab colors. Red = track parts, green = reward wells, blue = reward delivery, grey = DIO connections. Low opacity indicates part is not visible in picture from this viewing angle. Download Figure 4-1, TIF file. [file eneuro-12-ENEURO.0138-25.2025-s006.tif]
